# Prediction of Protein Structural Classes for Low-Similarity Sequences Based on Consensus Sequence and Segmented PSSM

**DOI:** 10.1155/2015/370756

**Published:** 2015-12-15

**Authors:** Yunyun Liang, Sanyang Liu, Shengli Zhang

**Affiliations:** School of Mathematics and Statistics, Xidian University, Xi'an 710071, China

## Abstract

Prediction of protein structural classes for low-similarity sequences is useful for understanding fold patterns, regulation, functions, and interactions of proteins. It is well known that feature extraction is significant to prediction of protein structural class and it mainly uses protein primary sequence, predicted secondary structure sequence, and position-specific scoring matrix (PSSM). Currently, prediction solely based on the PSSM has played a key role in improving the prediction accuracy. In this paper, we propose a novel method called CSP-SegPseP-SegACP by fusing consensus sequence (CS), segmented PsePSSM, and segmented autocovariance transformation (ACT) based on PSSM. Three widely used low-similarity datasets (1189, 25PDB, and 640) are adopted in this paper. Then a 700-dimensional (700D) feature vector is constructed and the dimension is decreased to 224D by using principal component analysis (PCA). To verify the performance of our method, rigorous jackknife cross-validation tests are performed on 1189, 25PDB, and 640 datasets. Comparison of our results with the existing PSSM-based methods demonstrates that our method achieves the favorable and competitive performance. This will offer an important complementary to other PSSM-based methods for prediction of protein structural classes for low-similarity sequences.

## 1. Introduction

Protein structural classes play a key role in protein science, simply because the biological function of a protein essentially related to its tertiary structure, which is determined by its amino acid sequence in accordance with the process of protein folding [1]. The knowledge of structural classes has been applied to reduce the search space of possible conformations of the tertiary structure [2, 3]; hence prediction of protein structural classes becomes a hot and challenging task in computational biology. The concept of protein structural classes was proposed by Levitt and Chothia [4], and a given protein can be categorized into mainly four structural classes according to the contents and spatial arrangements of the secondary structural elements of the protein domains; they are all-*α*, all-*β*, *α*/*β*, and *α* + *β*. The all-*α* and all-*β* proteins are mainly formed by helices and strands, respectively. The *α*/*β* protein mixes both helices and mostly parallel strands, and the *α* + *β* protein mixes both helices and mostly antiparallel strands.

During the last two decades, a great number of statistical learning algorithms had been developed to tackle this problem. Protein structural classes prediction is a typical pattern recognition problem, which is mainly performed in three steps. The first step is feature extraction, by which the different length sequences are converted into an equal length feature vectors. The methods include amino acid composition (AAC) [5–8], pseudoamino acid composition (PseAAC) [9–11], polypeptide composition [12, 13], functional domain composition [14], position-specific iterated-basic local alignment search tool (PSI-BLAST) profile [15–17], pseudo-position-specific scoring matrix (PsePSSM) [18, 19], and predicted protein secondary structure [20–22]. The second step is feature selection, which includes principal component analysis (PCA) [23], support vector machine-recursive feature elimination (SVM-RFE) [24], and wrapper and filter [25]. The final step is a choice of favorable classification algorithm. At present, the algorithms contain neural network [26], support vector machine (SVM) [27, 28], fuzzy clustering [29], Bayesian classification [30], rough sets [31], *k*-nearest neighbor [11], and so on. Among the three steps, feature extraction is the most critical step in this study for the successful improvement of protein structural classes prediction.

Currently, feature extraction methods mainly use protein primary sequence, predicted secondary structure sequence, and position-specific scoring matrix (PSSM). Position-specific scoring matrix can be obtained by giving a query sequence, which can be searched against a database of proteins using PSI-BLAST [32] and represents the evolutionary information. Recently, PSSM has attracted more attention and its prediction accuracy has been increasingly improved. AADP-PSSM [15] method extends the traditional dipeptide composition to PSSM. AAC-PSSM-AC [17] combines autocovariance and PSSM to extract the evolutionary information. AATP model [33] fuses AAC and transition probability composition from PSSM. In PSSS-PSSM [34], the predicted secondary structure information is employed to perform the prediction with evolutionary information. In MEDP [35], evolutionary difference formula is proposed based on PSSM. LCC-PSSM [25] extracts the long-range and linear correlation information from PSSM. PSSM-S [36] extracts the features relying on PSSM and proposes segmentation-based feature extraction technique based on the concepts of amino acids' distribution and autocovariance. The feature extraction methods relying on the position-specific scoring matrix (PSSM) have played a significant role to solve this classification issue. Though some of the existing methods have shown the excellent performance, the information embedded in the PSSM has not been adequately explored; there remains space for further improvement.

In this paper, we extract a consensus sequence based on PSSM, from which 40 global features are calculated. Then we propose two segmented feature extraction techniques based on the concepts of pseudo-position-specific scoring matrix (PsePSSM) and autocovariance transformation (ACT), which are defined on the PSSM, respectively. PsePSSM is originally proposed to avoid complete loss of the sequence-order information by Shen and Chou [18]. In other words, it reflects local information of PSSM. Autocovariance transformation as a statistical tool for analyzing sequences of vectors developed by Wold et al. [37]. ACT has been successfully used for protein pattern recognition [17, 38, 39], especially for protein classification, which is a correlation factor between two residues with a certain distance apart along a protein sequence. Hence, we obtain 380 segmented PsePSSM local features and 280 segmented ACT-PSSM local features. Finally, with the help of the three techniques, a 700D feature vector is constructed. In order to reduce the influence of redundancy, we use the principle component analysis (PCA) for feature selection. The 224 dominant features are selected for SVM classifier. To evaluate our method, jackknife cross-validation test is employed on three widely benchmark datasets; the experimental results show that our method is a state-of-the-art classifier and achieves the competitive performance compared with the other PSSM-based methods for low-similarity amino acid sequences.

## 2. Materials and Methods

### 2.1. Datasets

In order to facilitate the comparison with the previous works, three popular benchmark datasets are used to evaluate the performance of our method: the 1189 dataset [30], the 25*PDB* dataset [43], and the 640 dataset [44], which include 1092, 1673, and 640 protein domains with sequence similarity lower than 40%, 25%, and 25%, respectively. More details about the three datasets are listed in Table 1.

### 2.2. Feature Extraction

To develop a powerful predictor for the protein structural class based on position-specific scoring matrix (PSSM), the key is how to effectively define feature vectors to formulate the statistical samples concerned. Here, we use a combination of the consensus sequences, segmented PsePSSM, and segmented autocovariance transformation.

#### 2.2.1. Position-Specific Scoring Matrix

To extract the evolutionary information, we use each protein sequence (query sequence) as a seed to search and align homogenous sequences from NCBI's NR database (ftp://ftp.ncbi.nih.gov/blast/db) using the PSI-BLAST program [32] with parameters *h* = 0.001 and *j* = 3. PSI-BLAST will return a matrix; the (*i*, *j*)th entry of the obtained matrix represents the score of the amino acid residue in the *i*th position of the protein sequence being mutated to amino acid type *j* during the evolution process. The matrix is called the position-specific scoring matrix (PSSM) and it is denoted as

(1) $$\begin{array}{c} \text{PSSM}=\left(\;P_{1},P_{2},\ldots ,P_{j},\ldots ,P_{20}\;\right), \end{array}$$

where *P*_*j*_ = (*P*_1,*j*_, *P*_2,*j*_,…, *P*_*L*,*j*_)^*T*^,  (*j* = 1,2,…, 20). PSSM is a log-odds matrix of size *L* × 20, *L* represents the length of the query amino acid sequence and 20 is due to 20 amino acids, and *T* is the transpose operator. In this work, the PSSM elements are mapped to the range of [0,1] with the help of a standard sigmoid function:

(2) $$\begin{array}{c} f\left(\;x\;\right)=\frac{1}{\left(1+e^{-x}\right)}, \end{array}$$

where *x* is the original PSSM value.

#### 2.2.2. Consensus Sequence Based on PSSM

To extract global features, we adopt the method in [45, 46], which generates a consensus sequence (CS). It is constructed from PSSM as follows:

(3) $$\begin{array}{c} \alpha \left(\;i\;\right)=\arg\max\left\{\;P_{ij}\text{: }1\leq j\leq 20\;\right\},\;1\leq i\leq L, \end{array}$$

where “arg” represents the argument of the maximum. The *i*th base of the consensus sequence (CS) is then set to be the *α*(*i*)th amino acid in the amino acid alphabet and a consensus sequence is constructed. Next, we compute

(4) $$\begin{array}{c} \text{CSAAC}=\frac{n\left(j\right)}{L},\;1\leq j\leq 20, \end{array}$$

where *n*(*j*) represents the number of the amino acid *j* occurring in the consensus sequence. *L* represents the length of CS. Obviously, CSAAC represents 20 amino acid composition features of the CS.

Furthermore, we propose 20 composition moment features for CS, which have been applied for prediction of protein structural class mainly based on amino acid sequence [47] and predicted protein secondary structure sequence [34, 41]. They are formulated as

(5) $$\begin{array}{c} \text{CSCM}=\frac{\sum_{j=1}^{n_{i}}n_{ij}}{L\left(L-1\right)},\;1\leq i\leq 20,\;\;1\leq j\leq L, \end{array}$$

where *n*_*i*_ is the total number of the *i*th amino acid of 20 amino acids in the consensus sequence (CS) and *n*_*ij*_ represents the *j*th position in the CS (the length of *L*) of amino acid *i*.

In summary, we obtain 40 global features by combining 20 amino acid composition features with 20 composition moment features of CS-PSSM.

#### 2.2.3. PsePSSM Based on Segmented PSSM

To extract local features, we divide PSSM into *n* segments of equal length by applying a similar procedure in [46]. Let *L*_*i*_ = round(*L*/*n*); *L*_*i*_ represents the equal length except the last segment of the amino acid sequence; *i* represents the *i*th segment. However, the last segment may be longer or shorter owing to *L* not being always divisible by *n* and the last segment length can be *L* − ((*n* − 1)*∗L*_*i*_). Then, for each segment, we adopt the pseudo-PSSM (PsePSSM), which has been successfully applied to prediction of protein structural class [41]. Because the length of the shortest sequence of the three datasets is 10 (for 1189 dataset), hence *n* can be taken to only 2, 3, 4, and 5. However, if *n* = 4 or 5, the *λ* can be only equal to 1; this makes no meaning for the extracted features. So, *λ* is just taken to 2 and 3.

When *n* = 2, *L*_1_ = round(*L*/2); here we denote the length of the first segment sequence as *L*_1_ and the second segment sequence as *L*_2_ = *L* − *L*_1_, respectively. Hence, we obtain the segmented PsePSSM features according to the following equations:

(6) αjλ=1L1∑i=1L1Pi,j,j=1,2,…,20,  λ=0,1L1−λ∑i=1L1−λPi,j−Pi+λ,j2,j=1,2,…,20,  λ=1,2,3,4,βjλ=1L−L1∑i=L1+1LPi,j,j=1,2,…,20,  λ=0,1L−L1−λ∑i=L1+1L−λPi,j−Pi+λ,j2,j=1,2,…,20,  λ=1,2,3,4,

where *α*_*j*_^*λ*^ and *β*_*j*_^*λ*^ are the correlation factors of amino acid type *j*, respectively, whose contiguous distance is *λ* along each segmented protein sequence. Because the length of the shortest sequence of the three datasets is 10, when *n* = 2, the maximal value of parameter *λ* can be 4, so *λ* can be taken to 0, 1, 2, 3, and 4; here the 200 local features are obtained. Specially for *λ* = 0, *α*_*j*_^0^ and *β*_*j*_^0^ represent the average score of the amino acid residues in the two segmented protein *P* being mutated to amino acid type *j* during the evolution process.

When *n* = 3, *L*_1_ = round(*L*/3); here we denote the length of three segment sequences as *L*_1_, *L*_2_ = 2*L*_1_, *L*_3_ = *L* − 2*L*_1_. Hence, we obtain the segmented PsePSSM features, which can be defined by

(7) θjλ=1L1∑i=1L1Pi,j,j=1,2,…,20,  λ=0,1L1−λ∑i=1L1−λPi,j−Pi+λ,j2,j=1,2,…,20,  λ=1,2,μjλ=1L1∑i=L1+12L1Pi,j,j=1,2,…,20,  λ=0,1L1−λ∑i=L1+12L1−λPi,j−Pi+λ,j2,j=1,2,…,20,  λ=1,2,νjλ=1L−2L1∑i=2L1+1LPi,j,j=1,2,…,20,  λ=0,1L−2L1−λ∑i=2L1+1L−λPi,j−Pi+λ,j2,j=1,2,…,20,  λ=1,2,

where *θ*_*j*_^*λ*^, *μ*_*j*_^*λ*^, *ν*_*j*_^*λ*^ represent the same meaning as *n* = 2, respectively. When *n* = 3, the maximal *λ* can be equal to 2 and here we obtain 180 local features.

In the above-mentioned way, a total of 380 local features are extracted using segmented PsePSSM.

#### 2.2.4. Autocovariance Transformation Based on Segmented PSSM

In order to further obtain local features, here the autocovariance transformation (ACT) is introduced to get the neighboring effects of the sequences. The same as the previous section, we divide PSSM into *n* = 2 and *n* = 3 segments. Hence, we obtain the segmented ACT-PSSM features, which can be calculated by the following.

When *n* = 2,

(8) AC1jlg=1L1−lg∑i=1L1−lgPi,j−αj0Pi+lg,j−αj0,j=1,2,…,20,  lg=1,2,3,4,AC2jlg=1L−L1−lg∑i=L1+1L−lgPi,j−βj0Pi+lg,j−βj0,j=1,2,…,20,  lg=1,2,3,4.

When *n* = 3,

(9) AC1jlg=1L1−lg∑i=1L1−lgPi,j−θj0Pi+lg,j−θj0,j=1,2,…,20,  lg=1,2,AC2jlg=1L1−lg∑i=L1+12L1−lgPi,j−μj0Pi+lg,j−μj0,j=1,2,…,20,  lg=1,2,AC3jlg=1L−2L1−lg∑i=2L1+1L−lgPi,j−νj0Pi+lg,j−νj0,j=1,2,…,20,  lg=1,2,

where *lg* is the distance between two considered amino acid residues. Hence, a total of 280 local features are extracted using segmented ACT-PSSM.

To extract more global and local information from PSSM, we propose a comprehensive method called CSP-SegPseP-SegACP by fusing the 40 CS-PSSM features, the 380 segmented PsePSSM features, and the 280 segmented ACT-PSSM features. Finally, each protein sequence is characterized by a 700-dimensional (700D) feature vector.

### 2.3. Feature Selection

The dimension of our constructed feature vector is 700, which is a large input for SVM. The large dimension will lead to two problems: information redundancy or noise and dimension disaster. Hence, feature selection plays a key role in classification task. Principal component analysis (PCA) [23, 33] is one of the most classical dimensionality reduction method. The goal of PCA is to select some dominant features which can retain most of the information in terms of an orthogonal transformation; more details of PCA can be learned in the literature [48]. Finally, the 224 features are selected based on the 1189 dataset in the orthogonal space to perform the protein structural classes prediction.

### 2.4. Support Vector Machine

Support vector machine (SVM) is a well known machine learning algorithm based on statistical learning theory for binary classification problems, which is considered as the state-of-the-art classification technique and introduced by Vapnik in 1995 [49]. Protein structural class prediction is a four-classification problem, which can be converted into binary classification problem by using one against all strategy in this paper.

The basic idea of SVM is to find the separating hyperplane based on the support vector theory to minimize classification errors. It transforms the input data of samples to a higher dimensional space using the kernel function to find support vectors. Generally, four basic kernel functions are used by SVM, that is, linear function, polynomial function, sigmoid function, and radial basis function (RBF). Here, we choose the RBF as SVM's kernel due to its superiority for solving nonlinear problem [34, 46, 50], which is defined as *K*(*x*, *x*′) = exp⁡(−*γ*‖*x* − *x*′‖^2^). The kernel parameter *γ* and the cost parameter *C* are optimized based on the 1189 dataset by fifteenfold cross-validation using a grid search strategy in the LIBSVM package [51, 52], where *C* is allowed to take a value only between 2^−5^ and 2^15^ and *γ* only between 2^−15^ and 2^5^.

### 2.5. Performance Evaluation

Independent dataset test, subsampling test, and jackknife test are three widely used cross-validation methods in statistical prediction. Among these three methods, the jackknife test is deemed the most rigorous and objective due to its ability of yielding a unique result for a given dataset [53]. Hence, we adopt jackknife test in this study. During the process of the jackknife test, one protein sequence is singled out from the training set and the SVM classification model is trained by the remaining protein sequences. Then, the classification model is used to predict the singled out sequence. This process is repeated until every sequence in the training set has been singled out once. In this sense, the jackknife test is also known as the leave-one-out test.

To evaluate the performance of our method comprehensively, we report the seven standard performance measures, including sensitivity (Sens), specificity (Spec), *F*-measure, Matthew's correlation coefficient (MCC), Area Under ROC Curve (AUC), overall accuracy (OA), and average accuracy (AA). *F*-measure is a more robust metric by avoiding overestimating the performance of some metrics, which is the harmonic mean of recall and precision. MCC represents the correlation coefficients between the observed and the predicted class. Its value ranges from +1 (indicating best prediction model) to −1 (indicating worst prediction model). AUC is the area calculated under receiver operating characteristic (ROC) curve plotted by FP rate versus TP rate. Its value ranges from 0 to 1. These measures are defined as follows:

(10) Recall  or  Sens=TPTP+FN,Spec=TNFP+TN,Precision=TPTP+FP,F=2×Precision×RecallPrecision+Recall,MCC=TP×TN−FP×FNTP+FPTP+FNTN+FPTN+FN,AUC=12TPTP+FN+TNTN+FP,OA=TP+TNTP+FN+FP+TN,AA=∑Sensn,

where TP represents the number of true positives, FP represents the number of false positives, TN represents the number of true negatives, FN represents the number of false negatives, and *n* represents the number of classes, respectively.

## 3. Results and Discussion

**Figure 1:**
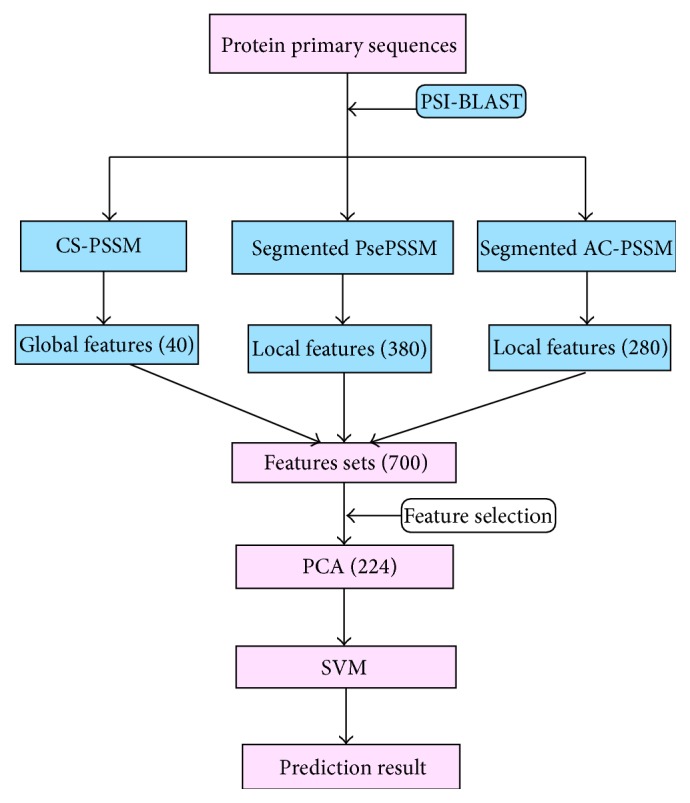
The flowchart of our proposed method.

In this study, a 700D feature vector is obtained and reduced to 224D by PCA to avoid dimension disaster. Then the 224 features are input into SVM. The RBF kernel function, the grid search approach, and the fifteenfold cross-validation for 1189 dataset are used to find the best parameters of *C* and *γ* for SVM. Finally, the optimal values of *C* and *γ* are computed to be 2 and 0.0019531, which are used in the experiments of Table 2 to avoid overfitting problem. To verify the performance of our method, rigorous jackknife cross-validation tests are performed on three widely used low-similarity datasets. The flowchart describes the whole process of the proposed method as shown in Figure 1.

### 3.1. Prediction Performance of Our Method

The overall protein structural class prediction accuracy (OA) as well as the prediction accuracy for each structural class has been achieved by using the combination of the features from the three sequence representation models, which include consensus sequence-PSSM (CSP), segmented PsePSSM, and segmented autocovariance transformation-PSSM (ACP). The proposed prediction method (CSP-SegPseP-SegACP) is examined with 1189, 25PDB, and 640 datasets by jackknife tests and we report the Sens, Spec, *F*-measure, MCC, and AUC for each structural class, the OA, as well as the AA. As listed in Table 2, relying solely on PSSM for feature extraction, we achieve up to 78.5%, 88.4%, and 77.0% overall accuracies for 1189, 25PDB, and 640 benchmark datasets, respectively, and average accuracies (AA) are also above 77.0% for three datasets. For 1189 and 640 datasets, through comparing the four structural classes with each other, the values of Sens, Spec, *F*-measure, MCC, and AUC in the all-*α* class, all-*β* class, and *α*/*β* class are obviously separately superior to those of *α* + *β* class. However, referring to the 25PDB dataset, *α* + *β* class obtains excellent performance for each performance measures; the prediction accuracy is up to 92.5%. For *α*/*β* class, the prediction accuracy is relatively low compared with the other classes. The fact indicates that there are still many difficulties to overcome in the future study to improve the prediction accuracies of *α*/*β* class and *α* + *β* class.

### 3.2. Performance Comparison between 224 Features and 700 Features

**Figure 2:**
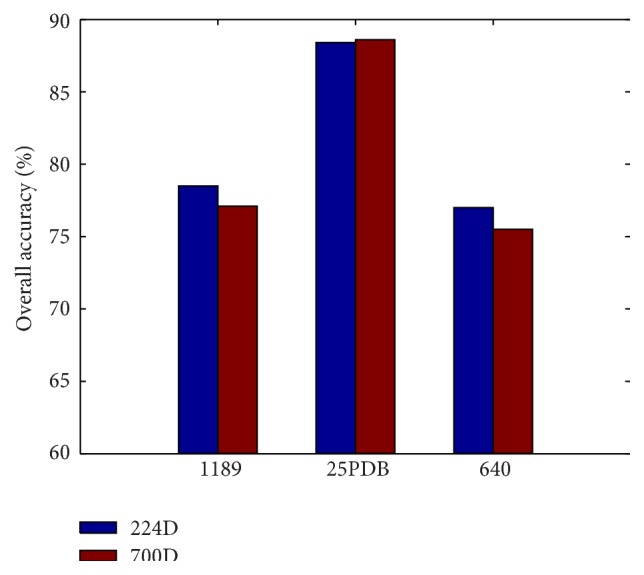
Comparison of accuracies between our method that includes 224 features and method that includes 700 features.

To overcome the impact of information redundancy and dimension disaster for SVM, the dimension of our obtained feature vector is reduced from 700 to 224 by using PCA. In this Section, we report the accuracies of our method using all 700 features on the three datasets, and we still optimize the SVM parameters *C* and *γ* on the 1189 dataset, which are computed to be 4 and 0.70711, respectively. The results are shown in Figure 2. The overall accuracies of 1189 and 640 datasets obtained by using 224 features both outperform those obtained by using 700 features, although the accuracy is 0.2% lower than that for 700D. The fact also fully shows that there indeed exists redundancy for SVM and PCA can retain the most dominant information in terms of an orthogonal transformation and save the calculation time at the same time.

### 3.3. Performance Analysis of Feature Groups

To investigate the contributions of feature groups on the protein structural class prediction accuracy, firstly, we calculate each feature group one by one on the 1189 dataset; the results are shown in Table 3. From Table 3, we can easily find that the best feature group is SegPseP, the second is segACP, and the last one is CSP. Moreover, by combination of each feature one by one, we calculate each combination group of features on the three datasets. As we can see from Table 4, each feature group makes a special contribution for the final prediction accuracy. Hence, we can summarize that features group SegPseP is optimal and plays an dominant role in improving the protein structural class prediction accuracies, especially for 25PDB dataset. Once again, it illustrates that the feature selection is the necessary step in this study.

### 3.4. Performance Comparison with Other Methods

In this section, to demonstrate the superiority of our method; the CSP-SegPseP-SegACP is further compared with the other recently reported prediction methods on the same datasets. We select the accuracy of each class and overall accuracy as evaluation indexes that are summarized in Table 5. The compared methods include other competitive PSSM-based methods such as PSSM-S [36], LCC-PSSM [25], MBMGAC-PSSM [40], RPSSM [34], AADP-PSSM [15], AAC-PSSM-AC [17], AATP [33], PsePSSM [41], Xia et al. [42], and MEDP [35], which are recently reported protein structural classes prediction methods based on the evolutionary information represented in the form of PSSM. MBMGAC-PSSM is our other method by fusing three autocorrelation descriptors and PSSM. RPSSM and PsePSSM are the submodels from PSSS-PSSM [34] and PSSS-PsePSSM [41], respectively.

As listed in Table 5, among these PSSM-based methods, our method achieves the competitive overall prediction accuracies for 1189, 25PDB, and 640 datasets. For 1189 dataset, the overall accuracies are separately 2.7% and 1.7% lower than the previous two better-performing results that are obtained by LCC-PSSM and PSSM-S methods. However, the overall accuracy for 1189 dataset outperforms the accuracies of the other seven PSSM-based methods. For 25PDB dataset, the OA is only 1.7% lower than the previous best-performing result that is calculated by PSSM-S method. For other nine PSSM-based methods, our method achieves the highest overall prediction accuracy with improvement of 9.4–27.6%. Referring to *α* + *β* class, our method achieves the highest result and the accuracy reaches 92.5%. For 640 dataset, although the OA is lower than that for LCC-PSSM and MBMGAC-PSSM, our method still obtains the satisfactory result. The facts sufficiently show that our proposed method successfully extracts the information hidden in the PSSM.

## 4. Conclusions

In this paper, the main contribution is to construct a 700D feature vector by three descriptors: consensus sequence- (CS-) PSSM, PsePSSM, and autocovariance transformation (ACT) based on segmented PSSM. While CS-PSSM reflects the global information, segmented PsePSSM and segmented ACT represent the local sequence-order information. Then 224 features are selected by using PCA. The SVM classifier and the jackknife test are employed to predict and evaluate the method on three benchmark datasets: 1189, 25PDB, and 640 datasets, with sequence similarity lower than 40%, 25%, and 25%, respectively. The experiment indicates that our approach can be used as a reliable tool and an excellent alternative for the accurate prediction of protein structural classes for low-similarity datasets. We shall make efforts in our future task to provide a public accessible web-server for the method presented in this paper. The codes are written in MATLAB language and can be downloaded from http://web.xidian.edu.cn/slzhang/paper.html.

## Figures and Tables

**Table 1:** The compositions of three datasets adopted in this paper.

Dataset	All-*α*	All-*β*	*α*/*β*	*α* + *β*	Total
1189	223	294	334	241	1092
25PDB	443	443	346	441	1673
640	138	154	177	171	640

**Table 2:** The prediction accuracies of our method on the 1189, 25PDB and 640 datasets.

Dataset	Structural class	Sens (%)	Spec (%)	*F*-measure	MCC	AUC
1189	All-*α*	84.8	95.6	0.84	0.80	0.90
	All-*β*	85.4	94.1	0.85	0.79	0.90
	*α*/*β*	85.0	90.0	0.82	0.74	0.88
	*α* + *β*	55.2	91.3	0.59	0.49	0.73
	OA	78.5				
	AA	77.6				

25PDB	All-*α*	94.4	96.4	0.92	0.90	0.95
	All-*β*	91.9	97.2	0.92	0.89	0.95
	*α*/*β*	71.1	95.7	0.76	0.70	0.83
	*α* + *β*	92.5	95.2	0.90	0.86	0.94
	OA	88.4				
	AA	87.5				

640	All-*α*	83.3	96.8	0.86	0.82	0.90
	All-*β*	83.1	95.3	0.84	0.79	0.89
	*α*/*β*	83.0	89.4	0.79	0.70	0.86
	*α* + *β*	60.2	87.4	0.62	0.49	0.74
	OA	77.0				
	AA	77.4				

**Table 3:** Performance comparison of our six feature groups on the 1189 dataset.

Dataset	Features	Prediction accuracy (%)				
		All-*α*	All-*β*	*α*/*β*	*α* + *β*	OA (%)
1189	CSAAC-PSSM (20D)	72.7	76.2	78.7	26.1	65.2
	CSCM-PSSM (20D)	69.1	76.9	82.0	29.9	66.5
	Seg2-PsePSSM (200D)	80.7	82.7	80.8	51.0	74.7
	Seg3-PsePSSM (180D)	79.8	80.6	81.4	48.1	73.5
	Seg2-ACPSSM (160D)	76.7	82.3	76.0	44.4	70.9
	Seg3-ACPSSM (120D)	69.1	77.6	78.4	38.6	67.5

**Table 4:** The contribution of each feature group for the overall accuracy (%).

Combination of feature groups	Dimension	1189	25PDB	640
CSAACP	20	65.2	62.0	66.0
CSAACP + CSCMP (CSP)	40	66.5	63.1	64.7
CSP + Seg2-PseP	240	75.2	74.4	75.8
CSP + Seg2-PseP + Seg3-PseP	420	76.2	87.7	74.5
CSP + SegPseP + seg2-ACP	680	76.1	87.9	75.0
CSP + SegPseP + seg2-ACP + seg3-ACP	700	77.1	88.6	75.5
CSP + SegPseP + SegACP-PCA	224	78.5	88.4	77.0

**Table 5:** Performance comparison of different methods on three datasets.

Dataset	Method	Prediction accuracy (%)				
		All-*α*	All-*β*	*α*/*β*	*α* + *β*	OA (%)
1189	PSSM-S [36]	93.3	85.1	77.6	65.6	80.2
	LCC-PSSM [25]	89.2	88.8	85.6	58.5	81.2
	MBMGAC-PSSM [40]	79.8	85.0	84.7	50.6	76.3
	RPSSM [34]	67.7	75.2	74.6	17.4	60.2
	AADP-PSSM [15]	69.1	83.7	85.6	35.7	70.7
	AATP [33]	72.7	85.4	82.9	42.7	72.6
	MEDP [35]	85.2	84.0	84.3	45.2	75.8
	PsePSSM [41]	82.0	82.3	84.1	44.0	74.4
	AAC-PSSM-AC [17]	80.7	86.4	81.4	45.2	74.6
	This paper	**84.8**	**85.4**	**85.0**	**55.2**	**78.5**

25PDB	PSSM-S [36]	93.8	92.8	92.6	81.7	90.1
	LCC-PSSM [25]	91.7	80.8	79.8	64.0	79.0
	MBMGAC-PSSM [40]	86.7	81.5	79.5	61.7	77.2
	RPSSM [34]	75.6	70.2	52.0	43.3	60.8
	AADP-PSSM [15]	83.3	78.1	76.3	54.4	72.9
	AATP [33]	81.9	74.7	75.1	55.8	71.7
	MEDP [35]	87.8	78.3	76.0	57.4	74.8
	AAC-PSSM-AC [17]	85.3	81.7	73.7	55.3	74.1
	PsePSSM [41]	86.2	78.8	75.7	57.6	75.5
	Xia et al. [42]	92.6	72.5	71.7	71.0	77.2
	This paper	**94.4**	**91.9**	**71.1**	**92.5**	**88.4**

640	LCC-PSSM [25]	92.8	88.3	85.9	66.1	82.7
	MBMGAC-PSSM [40]	86.2	83.1	85.3	63.2	79.1
	MEDP [35]	84.8	75.3	86.4	53.8	74.7
	PsePSSM [41]	73.9	76.6	85.3	51.5	71.7
	This paper	**83.3**	**83.1**	**83.0**	**60.2**	**77.0**
